# Dietary fibers with low hydration properties exacerbate diarrhea and impair intestinal health and nutrient digestibility in weaned piglets

**DOI:** 10.1186/s40104-022-00771-7

**Published:** 2022-11-09

**Authors:** Shuangbo Huang, Zhijuan Cui, Xiangyu Hao, Chuanhui Cheng, Jianzhao Chen, Deyuan Wu, Hefeng Luo, Jinping Deng, Chengquan Tan

**Affiliations:** 1 Guangdong Provincial Key Laboratory of Animal Nutrition Control, National Engineering Research Center for Breeding Swine Industry, Institute of Subtropical Animal Nutrition and Feed, College of Animal Science, South China Agricultural University, Guangzhou, 510642 Guangdong China; 2 Dekon Food and Agriculture Group, Chengdu, 610031 China; 3 Guangdong Laboratory for Lingnan Modern Agriculture, South China Agricultural University, Guangzhou, 510642 Guangdong China

**Keywords:** Diarrhea, Dietary fiber, Hydration property, Intestinal health, Nutrient digestibility, Weaned piglet

## Abstract

**Background:**

This study aimed to investigate the hydration properties of different-source fibrous materials by comparing their water-binding capacity (WBC), water swelling capacity (WSC), viscosity, and in vivo effects of selected samples on growth performance, nutrient digestibility, diarrhea, and intestinal health in weaned piglets.

**Methods:**

A total of 13 commercially available fibrous materials were first compared in chemical composition and in vitro hydration property. Subsequently, 40 weaned piglets were randomized to five experimental dietary groups (8 piglets per group): control diet (a basal diet without dietary fiber, CON), basal diet supplemented with 5% microcrystalline cellulose (MCC), 5% wheat bran (WB), 5% *Moringa* *oleifera* leaf powder (MOLP), or 5% sugar beet pulp (SBP), followed by analyzing their growth performance and diarrhea rate in a 28-d experiment. After the feeding experiment, anaesthetized piglets were killed, and their intestinal and colon content or plasma samples were analyzed in nutrient digestibility, intestinal morphology, intestinal barrier, short-chain fatty acids (SCFAs), and bacterial population.

**Results:**

In vitro studies showed low hydration properties for WB and MCC, while medium hydration properties for MOLP and SBP. In vivo studies indicated that compared with medium hydration property groups, low hydration property groups showed (1) exacerbated diarrhea, impaired intestinal health, and reduced apparent fecal digestibility of dry matter, gross energy, acid detergent fiber, and neutral detergent fiber; (2) decreased SCFAs concentration and relative levels of *Lactobacillus* and *Bifidobacterium*, but increased levels of *Escherichia coli* and *Brachyspira hyodysenteriae* in colon contents. Additionally, SBP showed optimal performance in reducing diarrhea and increasing SCFAs production. Correlation analysis revealed a positive correlation of fiber hydration properties with in vitro SCFAs production, and diarrhea index and nutrient digestibility were negatively and positively correlated with SCFAs levels in the colon contents of weaned piglets, respectively.

**Conclusions:**

Different-source dietary fibers varied in their hydration properties and impacts on diarrhea, microbial composition and SCFAs production in weaned piglets. WB and MCC could exacerbate diarrhea and impair nutrient digestibility, probably because their low hydration properties were detrimental to gut microbial homeostasis and fermentation. Our findings provide new ideas for rational use of fiber resources in weaned piglets.

**Supplementary Information:**

The online version contains supplementary material available at 10.1186/s40104-022-00771-7.

## Introduction

The period around weaning is generally characterized by intestinal dysfunction, diarrhea, and growth lag in weaned piglets [1]. Growing evidence showed that many fiber sources could help the piglets overcome the limitations of an impaired gastrointestinal tract [2–4]. However, not all fiber sources are suitable for weaned piglets with immature intestinal development, and the functional properties of fibrous sources are considered to be more important than the chemical composition (such as crude fiber) of the fibrous ingredients for the weaning diet [3, 5]. The polysaccharides that make up the cell wall and their intermolecular association are responsible for the functional properties of plant materials, including hydration properties (viscosity, water swelling capacity (WSC), water binding capacity (WBC)) and fermentability which are believed to determine the major functional properties of a dietary fiber. Although modern analytical techniques allow the quantification and characterization of the physical and chemical properties of dietary fiber in plant materials, few studies involved the utilization of these measured physicochemical properties to determine their functional effects (e.g., diarrhea, small intestinal transport time, or hindgut microbiota) in the gastrointestinal tract [6].

Accumulated evidence suggests that intervention of feed hydration properties through a specific dietary fiber can alter the intestinal health and nutrient digestion in weaned piglets [7]. For instance, highly viscous dietary fibers could limit the interaction of chyme with digestive enzymes in the gastrointestinal tract, thereby reducing nutrient digestion and absorption [5]. The dietary fiber with a high WBC is also shown to be unsuitable for weaned piglets due to its tendency to promote satiety and decrease feed intake [8–10]. Therefore, dietary fiber hydration properties are one of the important factors affecting its digestion and fermentation, allowing digestive and bacterial enzymes to interact with the fiber matrix, thus contributing to nutrient digestion [11]. Meanwhile, fibers with different hydration properties differ significantly in their effects on weaned piglets. Non-starch polysaccharides (NSP) with fermentable but different hydration properties, such as oligofructose and sugar beet pulp (SBP), vary in their effects on the intestinal flora of weaned piglets [12–14]. SBP rather than oligofructose provided the diets with the moderate amount of soluble dietary fiber promoting a beneficial shift in microbial colonization and reducing diarrhea. These findings demonstrated that selecting fibrous materials sources with suitable hydration properties could be an attractive strategy for regulating the intestinal health and diarrhea of weaned piglets. We hypothesize that the interventional effects of different sources of dietary fiber on weaned piglets might be related to their hydration properties and intestinal function. To our best knowledge, thus far, studies on the effects of dietary fibers with different hydration properties as a fiber source on nutrient digestibility and intestinal health in weaned piglets are limited.

In order to understand the relationship between fiber hydration properties and intestinal functions, different-source dietary fibers were used as models to mimic different hydration property fibers. Firstly, we measured the hydration properties of 13 commercially dietary fibers in vitro. Subsequently, the 4 selected fiber materials with different hydration properties were added separately into the weaning diet to study their effects on nutrient digestibility, diarrhea, intestinal health, and intestinal microbiota in weaned piglets. Finally, we analyzed the possible correlation of fermentability and hydration properties of the dietary fibers with diarrhea rate and nutrient digestibility in weaned piglets. This study aimed to explore the relationship between dietary fiber hydration properties and intestinal functions as well as the effects of selected dietary fibers on nutrient digestibility, diarrhea, intestinal health, and intestinal microbiota in weaned piglets.

## Materials and methods

### Fibrous ingredients

Thirteen fibrous ingredients were obtained from Joinsha Animal Health Products (Xiamen, Fujian, China). Carrageenan, guar gum and sodium alginate are typical colloidal-derived fiber ingredients. *Moringa* *oleifera* leaf powder (MOLP), *Broussonetia papyrifera* leaf powder, mulberry leaf powder, cassava residue and albumen mulberry powder are the characteristic feed resources in south China. SBP, a coproduct from commodity industries, represents a high-fiber ingredient with a high content of soluble fiber used in the feed industry [14]. Wheat bran (WB) is a conventional feed ingredient. Wheat aleurone, also known as the outer endosperm, is the innermost layer of wheat grain cortex. Microcrystalline cellulose (MCC) and wood spruce crude fiber concentrate represent the high-fiber ingredients with a high content of insoluble fiber [15].

### Proximate analysis of fibrous ingredients and diets

Fibrous ingredients and diets were dried at 60 °C for 4 d by placing them in a heat-drying room and keeping the moisture at less than 8%, followed by grinding them separately through a 1-mm screen, and analyzing dry matter (DM, AOAC method 930.15) and crude protein (CP, AOAC method 990.03) [16]. Crude fiber (CF), neutral detergent fiber (NDF), and acid detergent fiber (ADF) were obtained by the method of Van Soest [17]. All procedures were performed in duplicate and the results are displayed in Table 1.

**Table 1:** Chemical analysis of fibrous ingredients

Item	DM, % air-dried samples	CP, % DM	CF, % DM	NDF, % DM	ADF, % DM
MCC	98.5	0.3	49.4	89.0	82.6
Wheat aleurone	88.3	5.5	0.1	26.3	20.0
WB	85.5	15.6	5.1	41.2	11.5
Carrageenan	83.2	4.4	0.2	2.7	1.0
Albumen mulberry powder	93.5	25.7	13.5	20.1	15.6
Mulberry leaf powder	92.0	29.4	10.8	30.8	18.1
MOLP	93.7	26.4	27.4	34.0	17.4
Wood spruce crude fiber concentrate	95.2	13.7	35.1	68.2	43.2
SBP	87.0	7.7	20.9	48.2	27.0
*Broussonetia papyrifera* leaf powder	95.7	22.5	13.9	15.9	13.0
Guar gum	84.2	5.6	0.5	53.1	0.8
Sodium alginate	87.0	21.5	4.5	19.7	55.4
Cassava residue	87.5	10.8	23.8	56.6	45.2

*DM* dry matter, *CP* crude protein, *CF* crude fiber, *NDF* neutral detergent fiber, *ADF* acid detergent fiber, *MCC* microcrystalline cellulose. *WB* wheat bran, *MOLP* *Moringa* *oleifera* leaf powder, *SBP* sugar beet pulp. The results in the table are shown as the mean values of two replicates

### Hydration properties of fibrous ingredients

Hydration properties, including the water-binding capacity (WBC), water swelling capacity (WSC), and viscosity were determined as described with slight modification [18]. WBC was determined as previously described [19]. Briefly, dry fibrous ingredients (0.3 g) were weighed and left to stand in distilled water (10 mL) for 1 h at 25 °C, followed by centrifugation at 14,000 × *g* for 20 min, allowing the residues to stand for 30 min, drying overnight at 110 °C, and measuring the weight. The WBC was expressed as grams of water per gram of dry sample.

To determine the WSC, dry fibrous ingredients (0.2 g) were hydrated in 10 mL distilled water in a graduated test tube at room temperature for 18 h. The bed volume was recorded, and WSC was calculated by the equation: *WSC* (mL/g) = (*V*_*1*_ − *V*_*0*_)/*W*_*0*_, where *V*_*1*_, the volume of the hydrated fiber ingredients; *V*_*0*_, the volume of fiber ingredients prior to hydration; *W*_*0*_, the weight of fiber ingredients prior to hydration.

Viscosity was measured using a procedure modified after Serena and Bach Knudsen and was expressed in centipoise [20]. Briefly, dry fibrous ingredients (2 g) were dissolved in 10 mL of 0.9% NaCl solution and extracted in a water bath at 40 °C for 1 h, followed by centrifugation at 3500 × *g* for 25 min at 23 °C and removing 0.5 mL of the supernatant by suction. The shear viscosity of each suspension was measured using an Anton Paar MCR 102 rotational rheometer (Anton Paar GmbH, Graz, Austria) with the concentric cylinder geometry (28.907 mm measuring cup diameter, 26.663 mm bob diameter, and 40 mm gap length). Measurements were made at 25 °C, with shear rate varying from 0.1 to 100/s. Values were reported as the average shear rate of 225, 240, 255, 270, 285, and 300/s [21]. The viscosity of solution was measured at 25 °C.

### Analysis of in vitro fermentation and volatile fatty acids (VFAs) in fibrous ingredients

The 13 kinds of fibrous ingredients from different sources were incubated in duplicate using the in vitro two-stage procedures of enzyme incubation and dialysis. The samples were enzymatically hydrolyzed with pepsin and pancreatin, followed by in vitro fermentation of the enzymolyzed residues using a previously reported procedure [22, 23]. To reduce the variation between animals, the inoculum was prepared by mixing the caecum digesta of six newly slaughtered weaned male piglets (Duroc × Landrace × Yorkshire) for fermenting the 13 different fibrous ingredients. Caecum digesta samples were collected directly from the animals, and immediately placed in a pre-warmed CO_2_-filled container for transfer to the laboratory. Then, after weighing, each sample, an amount of pre-warmed (39 °C) anaerobic sterile saline (9 g/L NaCl) was added based on the combined weight, usually at a ratio of 1:5, followed by homogenizing the diluted material with a hand-mixer for 60 s and straining through a double layer of cheesecloth with 16 threads/cm in both directions. Next, the inoculum was dispensed into pre-warmed bottles containing substrate and medium, followed by in vitro fermentation of the enzymatically hydrolyzed residues using the procedure reported by Williams et al. [24]. Specifically, substrates (0.30 g) were weighed in duplicate in 150-mL screw cap bottles and flushed with CO_2_, followed by adding the fermentation medium with inoculum (60 mL) and incubating the mixture at 39 °C for 48 h. Meanwhile, two bottles without substrate were used as blanks. The gas produced during fermentation was measured by a pressure transducer (GP: 50, SIN-54978, Grand Island, NY, USA), equipped with a digital data tracker (Tracker 211, Intertechnology Inc., Canada). After each measurement, the bottles were vented, and fermentation was stopped at 48 h by quenching the bottles in iced water. The kinetics of fermentation were assessed by measuring cumulative gas production over time and gas accumulation was modeled as previously described [25].

At the end of fermentation, fermentation fluid was collected from the bottle for measurement of short-chain fatty acids (SCFAs: acetate, propionate, butyrate, and valerate) and branched-chain fatty acid (BCFAs: isobutyrate and isovalerate). The fresh fermentation fluid was pretreated, and SCFAs and BCFAs were extracted as follows [26]. Briefly, 1-mL fermentation fluid sample was mixed with 1 mL of ultra-pure water, followed by vortexing for 2 min, sonicating each sample in an ice bath for 10 min, and then centrifugation at 14,000 r/min for 10 min at 4 °C. Next, the supernatant was promptly transferred to a 2-mL centrifuge tube, and then supplemented with a total of 20 μL of 25% metaphosphoric acid solution and 0.25-g anhydrous sodium sulfate for acidification and salting out, respectively. After vortexing for 2 min, 1 mL of methyl tert-butyl ether was added, followed by further vortexing for 5 min, and centrifuging the supernatant at 14,000 r/min for another 10 min at 4 °C to remove precipitation. Finally, the upper extraction solution was harvested, filtered through a 0.22-µm Millipore pore membrane to a 2-mL sample vial, and stored at − 20 °C until gas chromatography–MS (GC–MS) analysis. All steps above were performed at 4 °C or on ice.

### Analysis of in vitro gas production parameters of fibrous ingredients

Gas accumulation curves were modeled using the mathematical model proposed by Tan et al. [27]. The cumulative gas production (*V*) was fitted to the biphasic model by the equation: *V* = *V*_*F*_ × (1 − exp (− *K* × *T*))/(1 + exp (*B* – *K* × *T*)), where *V*_*F*_ indicates the final asymptotic gas volume (mL/g); *K*, the fractional rate of gas production at a particular time point (h^−1^); *B*, a positive shape parameter without dimension; *FRD*_0_, the initial fractional rate of degradation of enzymolyzed residues of weaning diets at *t*-value = 0 (h^−1^), which was calculated by the following equation: *FRD*_0_ = *K* ÷ [1 + exp (*B*)]; the half-life (*T*_1/2_), the time at which half of the final gas production is generated, which was calculated by the following equations: *T*_1/2_ = ln [2 + exp(*B*)] ÷ *K*, with the subscripts following the factors in the biphasic model describing the fermented fraction (*V*_*F*_, *K*, *B*, *T*_1/2_, *FRD*_0_).

### Animals and experimental design

The management and design of the experiment followed the animal care rules approved by the South China Agricultural University Animal Care and Use Ethics Committee. The ethical approval number is 2022F173. The experimental regulations and methods were approved and then performed according to relevant criteria. A total of 40 healthy weaned piglets (Duroc × Landrace × Yorkshire crossbred male piglets) from 8 primiparous sows (8 sows with similar litter sizes, 5 piglets per litter) were allotted into five dietary groups based on their body weight. Five weaned piglets from the same litter were allocated to different treatment groups. The piglets were 28-day-old (weaned at 25-day-old and fed creep feed for 3-day after weaning), and their initial body weight was 7.39 ± 0.10 kg. Each dietary group contained 8 replicate pens, and piglets were raised individually in metabolic cages on five experimental diets: 5% MCC, 5% WB, 5% MOLP and 5% SBP (Table 2). The diets were formulated to meet the nutrient requirements for weaned piglets (NRC, 2012) [28]. We used ‘d’ to describe the trial time instead of weaning age throughout this study. This experiment lasted 28 d, and all the piglets received water and ration ad libitum.

**Table 2:** Composition of the experimental diets (as-fed basis)

Item^a^	CON	Low hydration properties		Medium hydration properties	
		MCC	WB	MOLP	SBP
Ingredients, %					
Corn	36.00	26.70	30.90	33.80	30.50
Expanded maize	20.00	20.00	20.00	20.00	20.00
Soybean meal (46%)	16.60	18.10	15.80	13.60	16.50
Fermented soybean meal	10.00	10.00	10.00	10.00	10.00
MCC	-	5.00	-	-	-
WB	-	-	5.00	-	-
MOLP	-	-	-	5.00	-
SBP	-	-	-	-	5.00
Whey powder (low protein)	6.50	6.50	6.50	6.50	6.50
Soybean oil	1.70	3.50	2.60	1.80	2.30
Sucrose	3.00	4.00	3.00	3.00	3.00
Glucose	1.50	1.50	1.50	1.50	1.50
Chloride choline (60%)	0.10	0.10	0.10	0.10	0.10
CaHPO_4_	1.05	1.05	1.05	1.05	1.05
Limestone	0.80	0.80	0.80	0.80	0.80
Sodium chloride	0.30	0.30	0.30	0.30	0.30
Zinc oxide	0.15	0.15	0.15	0.15	0.15
Antifungal agent	0.15	0.15	0.15	0.15	0.15
Citric acid	1.00	1.00	1.00	1.00	1.00
_ L_-Lysine HCI (98.5%)	0.50	0.50	0.50	0.60	0.50
_ DL_-Methionine (99%)	0.20	0.20	0.20	0.20	0.20
Threonine	0.20	0.20	0.20	0.20	0.20
Phytase	0.01	0.01	0.01	0.01	0.01
Vitamin premix^b^	0.04	0.04	0.04	0.04	0.04
Mineral premix^c^	0.20	0.20	0.20	0.20	0.20
Calculated composition					
DE, Mcal/kg	3.40	3.40	3.40	3.40	3.40
CP, %	18.50	18.50	18.50	18.50	18.50
EE, %	4.28	5.85	5.34	4.62	4.84
Ash, %	2.30	2.32	2.40	2.14	2.62
Ca, %	0.70	0.70	0.70	0.70	0.80
P, %	0.60	0.60	0.60	0.60	0.60
Lysine, %	1.40	1.40	1.40	1.40	1.40
Methionine, %	0.40	0.40	0.40	0.40	0.40
Analyzed composition^d^					
DM, %	96.68	96.42	97.56	95.97	96.80
GE, MJ/kg	17.11	17.29	17.29	17.13	17.13
CF, %	2.25	4.59	2.49	2.58	3.15
CP, %	18.55	18.32	18.73	18.44	18.45
NDF, %	7.95	11.43	10.69	8.10	8.85
ADF, %	4.85	9.56	6.10	5.27	5.66

^a^ *DE* digestible energy, *CP* crude protein, *EE* ether extract, *DM* dray matter, *GE* gross energy, *CF* crude fiber, *NDF* neutral detergent fiber, *ADF* acid detergent fiber, *CON* control diet group, *MCC* 5% microcrystalline cellulose diet group, *WB* 5% wheat bran diet group, *MOLP* 5% *Moringa oleifera* leaf powder diet group, *SBP* 5% sugar beet pulp diet group

^b^ Provided per kilogram of diet: 12,400 IU of vitamin A, 2800 IU of vitamin D_3_, 30 mg of vitamin E, 5 mg of vitamin K_3_, 3 mg of vitamin B_1_, 10 mg of vitamin B_2_, 8 mg of vitamin B_6_, 0.04 mg of vitamin B_12_, 40 mg of niacin, 15 mg of calcium pantothenate, 1 mg of folic acid and 0.08 mg of biotin

^c^ Provided per kg of complete diet: 120 mg of Zn, 120 mg of Fe, 16 mg of Cu, 70 mg of Mn, 0.70 mg of I, and 0.60 mg of Se

^d^ The results are shown as the mean values of two replicates

### Sample collection and processing

Individual pig body weight and feed disappearance were recorded to determine average daily gain (ADG), average daily feed intake (ADFI) and gain/feed ratio (G/F) on d 1, 14, 21 and 28 of the experiment. Fecal scores were monitored each morning during the feeding trial and quantified using a scale of 0 to 3, with 0 = solid, 1 = semi-solid, 2 = semi-liquid, and 3 = liquid. A piglet with a score greater than 1 was regarded as having diarrhea. Diarrhea rate (%) was calculated as a percentage of the number of diarrheal piglets during the trial period divided by the total number of piglets. Diarrhea index = sum of diarrhea scores of each group of piglets during the test period/(number of test days × number of piglets per group).

At the end of the feeding trial (d 28), 5 mL blood samples were collected into labeled heparinized tubes. Plasma samples were obtained by centrifuging the blood samples in labeled heparinized tubes at 3000 × *g* and 4 °C for 15 min and then stored at – 80 °C for further analysis.

Following blood collection, all piglets were euthanized, and fragments measuring 1 cm were sampled from duodenum (10 cm from pylorus), jejunum (mid-section), and ileum (5 cm to ileocecal junction) for morphological evaluation. Fragments of intestinal tissues were fixed in 4% paraformaldehyde for further histological evaluation. Briefly, the duodenum, jejunum and ileum specimens were dehydrated in a graded ethanol series, cleared with xylene, and embedded in paraffin, followed by preparing two pieces of 5 μm thick sections from the intestinal samples, staining them with hematoxylin–eosin, and observing them under an optical microscope. Ten fields were randomly selected to measure the villus height and the crypt depth, and calculate the villus height/crypt depth ratio (VCR), with the average of these values used for statistical analysis.

Additionally, 5-cm long intestinal segments were collected separately from duodenum (15 cm from pylorus), jejunum (mid-section), and ileum (10 cm to ileocecal junction). Subsequently, these segments were cut open, rinsed, dried, and weighed to calculate the intestinal weight per length. Meanwhile, contents (~ 10 g) (each content was divided into three samples) were collected from colon (5 cm from the junction of cecum and colon), snap-frozen in liquid nitrogen, and stored at – 80 °C for further analysis.

### Histopathological analysis

Histopathological analysis of fragments of intestinal tissues followed a previous method, and morphometry of intestinal villus and crypt was performed on an Olympus BX53 microscope (Olympus, Tokyo, Japan) equipped with the Axiovision software as previously described [29].

### Analysis of intestinal permeability-related plasma biomarkers

The plasma levels of diamine oxidase (MM-0438O1) and *D*-lactate (MM-77964O1) were detected using commercial ELISA kits (MEIMIAN, Yancheng, Jiangsu, China). Each sample was quantified in duplicate. ELISA assay was conducted based on the procedures as previously described [30]. Briefly, after standing at room temperature for at least 20 min, the kits were used for detection. The samples were placed into each well and incubated for 2 h, followed by adding biotin-antibody and incubation for another 1 h at 37 °C. Next, horseradish peroxidase-conjugate was immediately added into each well and incubated for 1 h, followed by incubation separately with chromogenic substrate and stop solution in the dark. Finally, the absorbance was measured with a microplate reader at 450 nm wavelength. The inter-assay and intra-assay coefficient of variation and detection range of the kits are shown in Additional file 1: Table S1.

### Analysis of the digestibility of energy and nutrients

From d 20 of the experiment, piglets in each group were fed their respective experimental diets plus 0.3% TiO_2_ as an exogenous marker. The diets containing 0.3% TiO_2_ were fed for a 5-d adjustment period (d 20 to 24), followed by a 3-d collection period (d 25 to 27). Fecal samples were collected twice daily at 07:00 and 15:30, pooled, placed in plastic bags, and stored at – 20 °C. After sample collection, the fecal samples from each piglet were thawed and pooled together, followed by drying in a forced-draft oven (65 °C) for 72 h, grinding through a 1-mm screen, and thorough mixing before collection of a subsample for chemical analysis. Chemical analysis of diets and freeze-dried feces was carried out as follows. Briefly, diets and feces were analyzed in terms of dry matter (DM, AOAC method 930.15) and CP (AOAC method 990.03) [16]. NDF and ADF were obtained by the method of Van Soest [17]. The gross energy (GE) in diets and feces was analyzed using an adiabatic oxygen bomb calorimeter (Parr Instruments, Moline, IL, USA). The diet and fecal samples were analyzed to establish the TiO_2_ content using a UV spectrophotometer as reported by Biasato et al. [31]. Based on the analyzed values of nutrient concentration, as well as the TiO_2_ concentration in the feed and feces, the nutrient digestibility was calculated by the following equation: apparent total tract digestibility (ATTD, %) = 100 – [100 × (*TT* × *FN*)/(*FT* × *TN*)] [32], with *TT* and *TN* for the TiO_2_ and nutrient concentrations (% DM) in a diet, and *FT* and *FN* for the TiO_2_ and nutrient concentrations (% DM) in feces, respectively.

### Analysis of SCFAs and BCFAs in colon contents

SCFAs and BCFAs in colon contents were analyzed by gas–liquid chromatography. The frozen content from the colon was thawed (0.2 g), followed by pretreating the sample and analysis by gas chromatography as described by Yang et al. [26].

### Fecal DNA Isolation and RT-qPCR

Three samples of colon contents were collected from each piglet. Bacterial DNA was extracted from the digesta samples (approximately 0.2 g) of colon using the E.Z.N.A stool DNA kit (Omega Bio-Tek, Doraville, GA, USA) according to the manufacturer's instructions. RT-qPCR analysis of the relative abundance of *Bifidobacterium*, *Enterococcus faecium*, *Lactobacillus*, *Escherichia coli*, *Salmonella*, and *Brachyspira hyodysenteriae* in all samples was performed by real-time PCR using SYBR Premix Ex Taq reagents (EZBioscience, Guangzhou, Guangdong, China). A reaction was run in a volume of 12 μL with 6 μL 2 × SYBR Green PCR Master Mix, 0.25 μL of each primer (100 nmol/L), and 5.5 μL template DNA. The universal bacterial reference primer set was selected for calculating the abundance of target bacterial, and the specific sequences are shown in Additional file 1: Table S2. The cycle procedure included 15 min at 95 ℃ and 49 cycles for 3 s at 95 ℃, 25 s at annealing temperature, and 60 s at 72 ℃. Each sample was run simultaneously in triplicate on the same PCR plate, and the average value of the number of copies was used for statistical analysis. The abundances of *Bifidobacterium*, *Enterococcus faecium*, *Lactobacillus*, and *Escherichia coli* were calculated as a relative value normalized to the total bacteria of the same sample. The abundances of *Salmonella* and *Brachyspira hyodysenteriae* were calculated by the RT-qPCR detection rate due to the failure of some samples to amplify the product.

### RT-qPCR analysis for gene expression

Total RNA from jejunum, ileum, or colon was extracted with the reagent box of Total RNA Kit as instructed by the manufacturer. The concentration of RNA was quantified using a NanoDrop®2000 (Thermo Fisher, Waltham, MA, USA). RNA samples with an A_260_/A_280_ ratio between 1.8 and 2.0 were considered of high quality. The integrity of RNA was measured by formaldehyde gel electrophoresis and the 28S:18S ribosomal RNA band ratio was determined as ≥ 1.8. After reverse transcription using Primer Script TM RT reagent Kit (EZBioscience, Guangzhou, Guangdong, China), RT-qPCR was performed to analyze the expression levels of zonula occludens-1 (*ZO-1*), zonula occludens-2 (*ZO-2*), claudin-4 (*CLDN-4*), occludin (*OCLN*), tumor necrosis factor-α (*TNF-α*), interleukin 1β (*IL-1β*), colony stimulating factor 3 (*CSF3*), and interleukin 10 (*IL-10*) on a Quant Studio 6 RealTime PCR System (Thermo Fisher, Waltham, MA, USA). Primer sequences are shown in Additional file 1: Table S2**.** The cycle procedure included a pre-cycling stage at 95 °C for 10 min, amplification at 95 °C for 15 s and 60 °C for 1 min for 40 cycles. Each target gene was individually normalized to the reference gene *β-actin* by using the quantification method of 2 ^−^^ΔΔct^.

### Statistical analysis

All analyses of hydration properties, in vitro gas production parameters, and SCFAs in fibrous ingredients were performed in duplicate. Four piglets were culled because of disease or death and were not included in the final data. A total of 36 piglets (the number of piglets is 7, 7, 7, 7 and 8 in CON, MCC, WB, MOLP, and SBP, respectively) completed the experiment and were used for data analysis, and an individual piglet was considered as an experimental unit in all statistical analyses. Before analysis, all data were tested for normality and homogeneity of variance using the Kolmogorov–Smirnov and Levene tests (with the significance level set at 5%) in SPSS 17.0. One-way ANOVA was used to determine the effects of five diets, and multiple comparisons were performed with Tukey's test. *P-*diet represents the *P* values between the five groups. Unpaired *t*-test or non-parametric test were used to determine the effects of WBC on dietary fiber. *P*-HP stands for *P* value between low hydration property groups and medium hydration property groups. Diarrhea rate and bacteria detection rate were analyzed using the Chi-square test. Data for diarrhea index, digestibility of ADF, weight of jejunum, and villus height of jejunum were not normally distributed and were analyzed using the Kruskal–Wallis test. Spearman’s correlational analyses were used to examine potential associations between in vitro hydration properties and fermentability of fibrous ingredients. Spearman’s correlational analyses were also used to examine potential associations between diarrhea index, ATTD, and SCFAs concentrations in colon contents of weaned piglets. The results are shown as mean or mean ± standard error of the mean (SEM) or Spearman correlation coefficient. Differences were considered as statistically significant at *P* < 0.05 and as a trend to significance at 0.05 ≤ *P* < 0.10.

## Results

### Proximate analysis of fibrous ingredients

Table 1 displays the analytical characterization of all tested samples in detail. The fiber content was seen to vary greatly in CF (from 0.1% DM for wheat aleurone to 49.4% DM for MCC), NDF (from 2.7% DM for carrageenan to 89.0% DM for MCC) and ADF (from 0.8% DM for guar gum to 82.6% DM for MCC). Meanwhile, CP was very low (from 0.3% DM for MCC to 29.4% DM for mulberry leaf powder).

### In vitro hydration properties of fibrous ingredients

Previous studies have reported different-source dietary fibers were used as models to mimic different physicochemical properties fibers, such as solubility or fermentability fibers [27, 33]. The results of this study also showed that there were significant differences in the hydration properties of fibers from different sources. The 13 fibrous ingredients also varied greatly in in vitro WBC, WSC, and viscosity (Table 3). In term of WBC, carrageenan had the highest value, and MCC had the lowest value. In term of WSC, carrageenan and sodium alginate were higher than the other fiber materials. In term of viscosity, guar gum, *Broussonetia* *papyrifera* leaf powder and mulberry leaf powder were significantly higher than the other 10 fiber materials. We note that these fibrous ingredients varied in the three different properties (WBC, WSC, and viscosity), and for convenience, the ingredients of the 13 fibers were classified into three levels based on their mean values of the three properties: low (WB, MCC, cassava residue, and wheat aleurone), medium (SBP, MOLP, wood spruce crude fiber concentrate, albumen mulberry powder, and mulberry leaf powder), and high (carrageenan, guar gum, sodium alginate, and *Broussonetia* *papyrifera* leaf powder). Numerically, the highest hydration properties were found for colloidal-derived fiber ingredients. Previous studies showed that dietary fibers with excessively high hydration properties, such as colloidal-derived fiber ingredients, were detrimental to intestinal function in weaned piglets [5, 8–10]. In this study, carrageenan, guar gum, sodium alginate, *Broussonetia* *papyrifera* leaf powder and mulberry leaf powder were too high in WBC, WSC or/and viscosity, so they were excluded in subsequent in vivo trials. MCC, WB, MOLP and SBP were selected for further feeding experiments, because WB and MCC had low hydration property (for convenience, we used ‘hydration property’ to describe WBC, WSC, and viscosity throughout this study), while MOLP and SBP had medium hydration property.

**Table 3:** In vitro viscosity, water-binding capacity (WBC), and water swelling capacity (WSC) of fibrous ingredients

Item	WBC, g/g	WSC, mL/g	Viscosity, mPa·s	Mean values of the three properties
Low hydration properties, 1.8–2.3				
MCC	2.1	1.2	2.0	1.8
Wheat aleurone	2.2	1.7	1.8	1.9
WB	2.3	2.1	1.8	2.1
Cassava residue	2.8	2.2	1.9	2.3
Medium hydration properties, 2.6–3.8				
Albumen mulberry powder	3.9	2.0	1.9	2.6
Mulberry leaf powder	3.5	2.0	2.4	2.6
MOLP	4.4	3.5	2.0	3.3
Wood spruce crude fiber concentrate	4.6	3.4	1.9	3.3
SBP	4.9	4.7	1.9	3.8
High hydration properties, 4.1–6.5				
*Broussonetia papyrifera* leaf powder	5.4	4.4	2.4	4.1
Guar gum	5.8	5.9	2.5	4.7
Sodium alginate	4.2	8.6	2.0	4.9
Carrageenan	8.4	9.2	1.9	6.5

*MCC* microcrystalline cellulose, *WB* wheat bran, *MOLP* *Moringa* *oleifera* leaf powder, *SBP* sugar beet pulp. The results in the table are shown as the mean values of two replicates

### Growth performance

The growth performance parameters of the test piglets are shown in Table 4. The inclusion of fibrous ingredients in CON diet showed no impact (*P* > 0.05) on the body weight, ADG and ADFI of piglets. Compared with CON group, SBP group showed a significant increase in G/F during d 1–14 (*P* < 0.05). Notably, compared with the medium hydration property groups, the low hydration property groups showed a downtrend (*P* = 0.07) in G/F during d 1–14.

**Table 4:** Effects of dietary fibers with different hydration properties on the growth performance of weaned piglets

Item	CON	Low hydration properties		Medium hydration properties		SEM	*P*-value	
		MCC	WB	MOLP	SBP		*P*-diet	*P*-HP
No. of piglets	7	7	7	7	8			
Body weight, kg								
Initial weight	7.41	7.46	7.36	7.43	7.40	0.11	0.99	0.99
On d 14	12.32	11.81	12.04	11.55	12.37	0.28	0.89	0.92
On d 21	15.54	15.05	15.59	15.20	15.82	0.38	0.97	0.79
On d 28	18.53	18.77	19.28	18.43	19.37	0.41	0.94	0.91
ADG, g/d								
D 1 to 14	350.70	310.41	334.29	294.10	355.63	14.39	0.63	0.89
D 1 to 21	387.35	361.22	392.11	370.00	401.31	14.27	0.91	0.73
D 1 to 28	397.09	403.88	425.82	392.70	427.68	11.87	0.84	0.88
ADFI, g/d								
D 1 to 14	516.41	493.19	493.53	473.71	508.03	16.60	0.95	0.97
D 1 to 21	591.74	576.81	589.30	588.44	603.08	16.93	0.99	0.71
D 1 to 28	656.49	653.10	670.51	673.36	681.40	16.93	0.99	0.64
G/F ratio								
D 1 to 14	0.66^a^	0.63^a^	0.63^a^	0.61^a^	0.72^b^	0.01	< 0.01	0.07
D 1 to 21	0.65	0.62	0.66	0.63	0.66	0.01	0.71	0.78
D 1 to 28	0.60	0.62	0.63	0.58	0.63	0.01	0.21	0.16

*CON* control diet group, *MCC* 5% microcrystalline cellulose diet group, *WB* 5% wheat bran diet group, *MOLP* 5% *Moringa* *oleifera* leaf powder diet group, *SBP* 5% sugar beet pulp diet group. *ADG* average daily gain, *ADFI* average daily feed intake, *G/F* gain: feed. ^a,b^ Different lowercase letters represent significant difference at *P* < 0.05, and *P*-diet represents the *P* values between the five groups. *P*-HP stands for *P* value between low hydration property groups and medium hydration property groups

### Diarrhea rate and diarrhea index

In Table 5, the five groups showed significant differences in diarrhea rate during the whole experimental period (*P* < 0.01). Compared with CON group, MCC and WB groups showed an uptrend (0.05 < *P* < 0.10) in diarrhea rate during d 1–7 and 1–14, with a significantly higher (*P* < 0.05) diarrhea rate during d 1–21 and 1–28. Meanwhile, MOLP group showed a higher (*P* < 0.05) diarrhea rate during d 1–7, in contrast to a lower (*P* < 0.05) diarrhea rate for SBP group during d 1–7 and a downtrend (0.05 < *P* < 0.10) during d 1–14.

**Table 5:** Effects of dietary fibers with different hydration properties on the diarrhea rate and index of weaned piglets

Item	CON	Low hydration properties		Medium hydration properties		SEM	*P*-value	
		MCC	WB	MOLP	SBP		*P*-diet	*P-*HP
No. of piglets	7	7	7	7	8			
Diarrhea rate, %								
D 1 to 7	8.16^b^	14.29^bc^	12.25^bc^	18.37^c^	1.79^a^	1.20	< 0.01	0.15
D 1 to 14	5.10^ab^	14.29^b^	14.29^b^	9.18^ab^	3.57^a^	0.82	< 0.01	0.10
D 1 to 21	4.08^a^	10.88^b^	10.88^b^	6.10^a^	4.17^a^	0.59	< 0.01	0.03
D 1 to 28	4.59^a^	8.16^b^	8.16^b^	4.59^a^	3.57^a^	0.66	< 0.01	0.04
Diarrhea index								
D 1 to 7	0.15	0.27	0.28	0.35	0.11	0.05	0.46	0.39
D 1 to 14	0.13	0.31	0.30	0.21	0.13	0.05	0.90	0.06
D 1 to 21	0.12	0.26	0.26	0.16	0.12	0.04	0.95	0.04
D 1 to 28	0.13	0.21	0.19	0.14	0.12	0.03	0.85	0.30

*CON* control diet group, *MCC* 5% microcrystalline cellulose diet group; WB, 5% wheat bran diet group, *MOLP* 5% *Moringa* *oleifera* leaf powder diet group, *SBP* 5% sugar beet pulp diet group

^a−c^ Different lowercase letters represent significant difference at *P* < 0.05, and *P-*diet represents the *P* values between the five groups. *P*-HP stands for *P* value between low hydration property groups and medium hydration property groups. Diarrhea rate was calculated as a percentage of the number of diarrheal piglets during the period divided by the total number of piglets. Diarrhea index = sum of diarrhea scores of each group of piglets during the test period/(number of test days × number of piglets per group)

Note that the different hydration property groups showed significant differences in diarrhea rate. Compared with medium hydration property groups, low hydration property groups showed a significant increase (*P* < 0.05) in diarrhea rate during d 1–21 and d 1–28, and diarrhea index during d 1–21.

### Intestinal weight per length and intestinal morphology

In Table 6, the five groups were seen to have no significant difference (*P* > 0.05) in intestinal weight per length, villus height, and crypt depth of duodenum, jejunum, and ileum. Moreover, the hydration properties of dietary fibers did not affect (*P* > 0.05) intestinal weight per length and intestinal morphology. However, MCC group with the poorest hydration properties showed lower (*P* < 0.05) villus height/crypt depth ratio (VCR) in jejunum when compared to the other four groups.

**Table 6:** Effects of dietary fibers with different hydration properties on the intestinal morphology of weaned piglets

Item	CON	Low hydration properties		Medium hydration properties		SEM	*P*-value	
		MCC	WB	MOLP	SBP		*P*-diet	*P-*HP
No. of piglets	7	7	7	7	8			
Duodenum								
Weight, g/cm	0.8	0.9	1.0	0.8	0.9	0.02	0.09	0.85
Villus height, μm	384.6	400.3	388.3	417.6	380.9	11.90	0.88	0.89
Crypt depth, μm	164.2	178.1	171.8	180	162.1	8.45	0.96	0.81
VCR	2.7	2.9	2.6	2.8	2.8	0.10	0.98	0.95
Jejunum								
Weight, g/cm	0.5	0.5	0.5	0.5	0.4	0.03	0.82	0.15
Villus height, μm	378.9	363.3	361.3	373.2	376.8	8.51	0.96	0.50
Crypt depth, μm	159.0	176.5	146.9	166.1	161.3	4.60	0.40	0.87
VCR	2.8^a^	2.2^b^	2.8^a^	2.7^a^	2.8^a^	0.10	0.04	0.21
Ileum								
Weight, g/cm	0.7	0.7	0.8	0.8	0.7	0.04	0.92	0.88
Villus height, μm	273.4	283.5	283.2	294.7	272.7	8.82	0.94	0.99
Crypt depth, μm	136	148.4	147.9	161.4	145.2	6.34	0.82	0.75
VCR	2.4	2.4	2.4	2.4	2.3	0.08	0.99	0.77

*CON* control diet group, *MCC* 5% microcrystalline cellulose diet group, *WB* 5% wheat bran diet group, *MOLP* 5% *Moringa* *oleifera* leaf powder diet group, *SBP* 5% sugar beet pulp diet group. VCR, villus height:crypt depth ratio

^a,b^ Different lowercase letters represent significant difference at *P* < 0.05, and *P-*diet represents the *P* values between the five groups. *P*-HP stands for *P* value between low hydration property groups and medium hydration property groups

### Digestibility of energy and nutrients

As shown in Table 7, MCC and WB groups were significantly lower than the other three groups in ATTD of DM, GE, and NDF (*P* < 0.05). Compared with medium hydration property groups, low hydration property groups showed a significant decrease in ATTD of DM, GE, NDF, and ADF (*P* < 0.05).

**Table 7:** Effects of dietary fibers with different hydration properties on the apparent total tract digestibility (ATTD) of dry matter (DM), gross energy (GE), crude protein (CP), neutral detergent fiber (NDF), and acid detergent fiber (ADF) in weaned piglets

Item	CON	Low hydration properties		Medium hydration properties		SEM	*P*-value	
		MCC	WB	MOLP	SBP		*P*-diet	*P-*HP
No. of piglets	6	6	6	6	7			
Digestibility, %								
DM	90.2^b^	79.5^a^	83.3^a^	90.2^b^	89.4^b^	1.04	< 0.01	< 0.01
GE	89.5^b^	79.2^a^	82.3^a^	90.6^b^	88.6^b^	1.03	< 0.01	< 0.01
CP	82.0	84.1	84.4	85.2	83.2	1.03	0.69	0.93
NDF	62.3^b^	45.4^a^	47.4^a^	61.3^b^	61.7^b^	2.34	0.01	< 0.01
ADF	62.0^b^	43.0^a^	49.7^ab^	64.9^b^	67.0^b^	2.44	< 0.01	< 0.01

*CON* control diet group, *MCC* 5% microcrystalline cellulose diet group, *WB* 5% wheat bran diet group, *MOLP* 5% *Moringa* *oleifera* leaf powder diet group, *SBP* 5% sugar beet pulp diet group. The recovery rate of TiO_2_ in the feces of five piglets was too low to be included in the statistics

^a,b^ Different lowercase letters represent significant difference at *P* < 0.05, and *P*-diet represents the *P* values between the five groups. *P*-HP stands for *P* value between low hydration property groups and medium hydration property groups

### Intestinal barrier and inflammatory cytokines

**Fig. 1:**
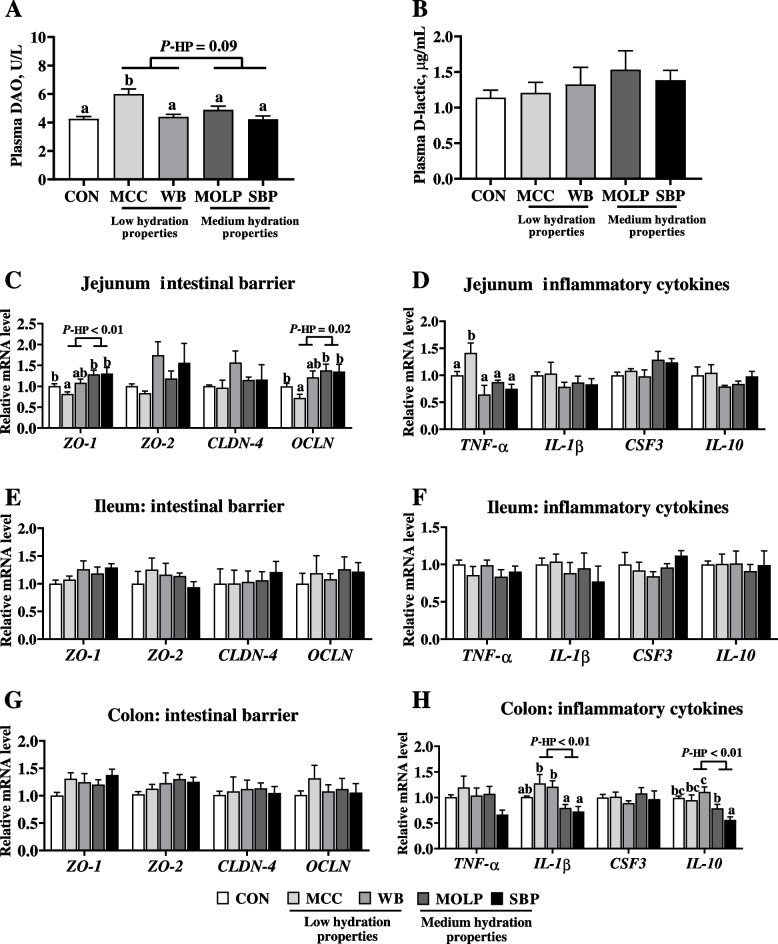
Effects of dietary fibers with different WBC on the intestinal barrier and inflammatory cytokines of weaned piglets. (A, B) Plasma diamine oxidase and *D*-lactate, intestinal permeability-related plasm biomarkers. (C-H) The mRNA expression of intestinal barrier and cytokine related genes in jejunum, ileum, and colon of weaned piglets. CON, control diet group; MCC, 5% microcrystalline cellulose diet group; WB, 5% wheat bran diet group; MOLP, 5% *Moringa* *oleifera* leaf powder diet group; SBP, 5% sugar beet pulp diet group; ZO-1, zonula occludens-1; ZO-2, zonula occludens-2; CLDN-4, claudin-4; OCLN, occludin; TNF-α, tumor necrosis factor-α; IL-1β, interleukin 1β; CSF3, colony stimulating factor 3; IL-10, interleukin 10. ^a−c^Bars with different lowercase letters differ significantly (*P* < 0.05), and *P-*diet represents the *P* values between the five groups. *P*-HP stands for *P* value between low hydration property groups and medium hydration property groups. Values are presented as means ± Pooled SEM

In Fig. 1A, DAO level was seen to be significantly higher in MCC group than in the other four groups (*P* < 0.05). MCC group showed an increase in the *TNF-α* mRNA level, but a decrease (*P* < 0.05; Fig. 1C, D) in the *ZO-1* and *OCLN* mRNA levels in jejunum relative to the other four groups. However, the five groups showed no difference (*P* > 0.05; Fig. 1E, F) in the mRNA levels of intestinal barrier and inflammatory cytokines-related genes in ileum. Moreover, compared with medium hydration property groups, low hydration property groups showed significantly lower mRNA levels of *ZO-1* and *OCLN* in jejunum, and higher mRNA levels of *IL-1β* and *IL-10* in colon (*P* > 0.05; Fig. 1C, H).

### Bacterial populations in colon contents

Dietary fiber inclusion did not affect total bacteria in colon of the piglets (data not shown). In Table 8, compared with CON group, SBP group showed higher relative abundances of *Lactobacillus* and *Bifidobacterium*, but a lower qPCR detection rate of *Brachyspira hyodysenteriae* (*P* < 0.05). Interestingly, compared with medium hydration property groups, low hydration property groups showed notably lower relative abundances of *Lactobacillus* and *Bifidobacterium*, but higher relative abundances of *Escherichia coli* and qPCR detection rate of *Brachyspira hyodysenteriae* (*P* < 0.05).

**Table 8:** Concentrations of short-chain fatty acids (SCFAs), branched-chain fatty acids (BCFAs) and bacterial population in the colon contents of different groups

Item	CON	Low hydration properties		Medium hydration properties		SEM	*P*-value	
		MCC	WB	MOLP	SBP		*P*-diet	*P-*HP
No. of piglets	7	7	7	7	8			
Bacterial population, relative abundance of CON								
* Lactobacillus*	1.00^a^	1.11^a^	1.20^a^	1.29^a^	2.44^b^	0.28	0.02	< 0.01
* Bifidobacterium*	1.00^ab^	0.79^a^	1.31^ab^	1.55^c^	2.84^d^	0.30	0.01	< 0.01
* Enterococcus faecium*	1.00	2.10	1.68	1.60	1.51	0.20	0.66	0.54
* Escherichia coli*	1.00^abc^	1.41^bc^	1.42^c^	0.68^ab^	0.40^a^	0.15	0.01	< 0.01
Detection rate of bacteria, %								
* Salmonella*	62.50	75.00	87.50	75.00	50.00		0.19	0.65
* Brachyspira hyodysenteriae*	62.50^a^	62.50^a^	50.00^a^	37.50^a^	6.25^b^		< 0.01	< 0.01
Concentration of VFA, mg/g (dry matter of colon content)								
Acetate	1.58	1.21	1.31	2.16	2.33	0.11	0.06	< 0.01
Propionate	1.13	0.94	0.90	1.22	1.71	0.09	0.35	< 0.01
Butyrate	0.64^b^	0.39^a^	0.56^ab^	0.61^bc^	0.79^c^	0.04	< 0.01	< 0.01
Valerate	0.23^a^	0.19^a^	0.25^a^	0.30^ab^	0.38^b^	0.02	< 0.01	0.04
Total SCFA	3.58^a^	2.73^a^	3.03^a^	4.29^ab^	5.20^c^	0.22	< 0.01	< 0.01
Isobutyrate	0.15	0.12	0.13	0.18	0.21	0.01	0.14	< 0.01
Isovalerate	0.24	0.26	0.27	0.32	0.38	0.02	0.06	< 0.01
Total BCFA	0.39	0.38	0.40	0.50	0.59	0.03	0.13	0.01
Total VFA	3.96^ab^	3.11^a^	3.43^a^	4.79^bc^	5.78^c^	0.24	< 0.01	< 0.01
SCFA/VFA, %	90.17	87.75	88.43	89.43	89.75	0.42	0.34	0.04
BCFA/VFA, %	9.83	12.25	11.57	10.57	10.94	0.43	0.34	0.04

*CON* control diet group, *MCC* 5% microcrystalline cellulose diet group, *WB* 5% wheat bran diet group, *MOLP* 5% *Moringa* *oleifera* leaf powder diet group, *SBP* 5% sugar beet pulp diet group, *VFA* volatile fatty acid. The abundances of *Bifidobacterium*, *Enterococcus faecium*, *Lactobacillus*, *Escherichia coli* were calculated as a relative value normalized to the total bacteria of the same sample. The abundances of *Salmonella* and *Brachyspira hyodysenteriae* were calculated by the detection rate due to the failure of some samples to amplify the product; total SCFA, the sum of acetate, propionate, butyrate, and valerate. Total BCFA, isobutyrate and isovalerate; total VFA, the sum of acetate, propionate, butyrate, isobutyrate, valerate and isovalerate. Dry matter is 257, 268, 278, 240, and 245 g/kg in colon contents of piglets in CON, MCC, WB, MOLP, and SBP, respectively

^a−d^ Different lowercase letters represent significant difference at *P* < 0.05, and *P-*diet represents the *P* values between the five groups. *P*-HP stands for *P* value between low hydration property groups and medium hydration property groups

### SCFAs and BCFAs in colon contents

A growing body of evidence supports that fermentable fibers and proteins influence intestinal health through production of SCFAs or BCFAs [34, 35]. In this study, we investigated the relationship of dietary fiber hydration properties with SCFAs and BCFAs production in colon of piglets. As shown in Table 8, compared with CON group, SBP group showed higher (*P* < 0.05) levels of butyrate, valerate, total SCFA, and total volatile fatty acid (total VFA, the sum of acetate, propionate, butyrate, isobutyrate, valerate and isovalerate). Besides, we noted that the two low hydration property dietary fibers were lower than the two medium hydration property dietary fibers in SCFAs production capacity, but higher in BCFAs production. Compared with medium hydration property groups, low hydration property groups showed lower (*P* < 0.05) SCFAs/VFAs, and higher (*P* < 0.05) BCFAs/VFAs.

### In vitro fermentability of fibrous ingredients

**Fig. 2:**
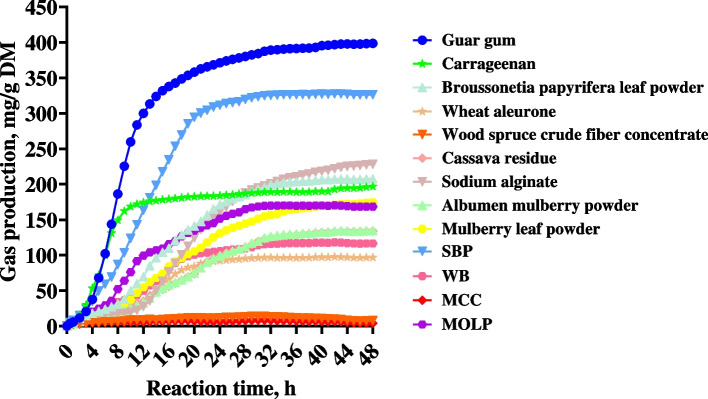
Gas production curves of in vitro fermentation of fibrous ingredients. DM, dry matter; MCC, microcrystalline cellulose; WB, wheat bran; MOLP, *Moringa* *oleifera* leaf powder; SBP, sugar beet pulp. Values are presented as the mean values of two replicates

Table 9 and Fig. 2 show the gas production parameters and VFAs levels during in vitro fermentation of the 13 fibrous ingredients from different sources, and they were seen to vary in the test parameters. Previous studies have shown that VFAs production parameters were more correlated with weaning piglet performance than gas production parameters [36]. In this study, we focused on the VFAs production parameters of fibrous ingredients. Guar gum, SBP, sodium alginate, and *Broussonetia* *papyrifera* leaf powder produced higher concentrations of acetate and total VFAs than the other fibrous ingredients, indicating that their fermentation capacity was higher. Notably, in terms of gas production parameters and the ability to produce VFAs, WB and MCC (the two fibrous ingredients with a low hydration property) were lower than SBP and MOLP (the two fibrous ingredients with a medium hydration property) (SBP > MOLP > WB > MCC).

**Table 9:** Gas production parameters and concentrations of volatile fatty acids (VFAs) during in vitro fermentation of enzymatically hydrolyzed residues of the 13 fibrous ingredients using cecum digesta from weaned piglets

Item	Gas production parameters						Concentrations of VFAs, mmol/L							
	V, mL/g	V_F_, mL/g	(FRD_0_) × 100, h^−1^	K, h^−1^	T_1/2_, h	Acetate	Propionate	Butyrate	Valerate	Isobutyrate	Isovalerate	Total SCFA	SCFAs/VFAs	BCFAs/ VFAs
Guar gum	398.6	385.4	2.3	0.3	8.5	27.8	18.8	3.8	1.3	0.5	0.6	52.9	97.7	2.1
SBP	326.2	327.7	1.5	0.2	12.0	29.2	9.5	2.9	1.3	0.3	0.4	43.4	98.8	1.6
Sodium alginate	228.1	222.0	0.5	0.2	19.2	30.7	7.5	1.9	1.2	0.4	0.3	42.0	98.3	1.7
*Broussonetia papyrifera* leaf powder	207.8	209.0	1.3	0.2	15.5	25.9	7.2	2.0	1.3	0.5	0.7	37.5	97.1	3.2
Carrageenan	172.7	168.0	2.2	0.5	6.1	19.2	10.8	3.1	1.4	0.4	0.6	35.6	96.9	2.8
MOLP	167.5	170.6	2.8	0.2	11.7	21.4	6.0	1.9	1.2	0.4	0.6	31.6	96.5	3.2
Mulberry leaf powder	174.5	175.7	1.4	0.1	17.1	21.4	6.5	1.5	1.2	0.4	0.6	31.5	97.1	3.2
Albumen mulberry powder	135.0	137.8	1.0	0.2	18.2	19.4	5.7	1.4	1.2	0.4	0.6	28.7	96.5	3.5
Cassava residue	134.1	139.1	1.4	0.1	18.0	17.3	5.9	1.3	1.2	0.3	0.5	26.5	97.0	3.0
WB	116.6	118.7	2.2	0.2	12.4	15.4	5.9	2.1	1.2	0.4	0.5	25.4	96.9	3.5
Wheat aleurone	96.8	98.5	1.2	0.2	13.3	13.8	5.2	2.1	1.2	0.4	0.6	23.2	96.1	4.3
MCC	4.2	6.9	30.5	0.3	2.8	6.0	1.8	0.4	0.9	0.3	0.4	9.9	91.9	7.1
Wood spruce crude fiber concentrate	7.9	11.2	10.7	0.2	5.4	6.1	1.8	0.4	0.9	0.2	0.4	9.8	93.9	6.1

*SBP* sugar beet pulp, *MOLP* *Moringa* *oleifera* leaf powder, *WB* wheat bran, *MCC* microcrystalline cellulose, *V* 48 h cumulative gas production, *V*_*F*_ the final asymptotic gas volume, *FRD*_*0*_ initial fractional rate of degradation at *t*-value = 0; *K*, fractional rate of gas production at a particular time point; *T*_*1/2*_, half-life to asymptote; total SCFA, the sum of acetate, propionate, butyrate, and valerate; SCFAs/VFAs, the sum of SCFAs (acetate, propionate, butyrate, and valerate)/the sum of VFAs; BCFAs/VFAs, the sum of BCFAs (isobutyrate and isovalerate)/the sum of VFAs; the results in the table are shown as the means values of two replicates

### Spearman correlations between in vitro hydration properties and fermentability of fibrous ingredients

Previous studies have shown the physicochemical characteristics of a fibrous ingredient as a key factor influencing its fermentability, such as solubility, viscosity, and accessibility [6]. We analyzed the Spearman correlation coefficient between in vitro hydration properties and fermentation parameters of fibrous ingredients (Table 10). Interestingly, Spearman correlation analysis revealed a correlation of WBC with V, V_F_, acetate, propionate, valerate, total SCFA, SCFAs/VFAs, and BCFAs/VFAs (*r* = 0.68, 0.64, 0.57, 0.74, 0.58, 0.72, 0.68, 0.50, and –0.59, respectively; *P* < 0.05). WSC also showed a correlation with V, V_F_, acetate, propionate, valerate, total SCFAs, SCFAs/VFAs, and BCFAs/VFAs (*r* = 0.67, 0.65, 0.77, 0.66, 0.68, 0.66, and –0.76, respectively; *P* < 0.05). Moreover, viscosity was positively correlated with V, V_F_, total SCFAs, and SCFAs/VFAs (*r* = 0.55, 0.58, 0.54, and 0.40, respectively; *P* < 0.05).

**Table 10:** Spearman correlations between in vitro hydration properties and fermentability of the 13 fibrous ingredients

Spearman correlation	WBC, g/g	WSC, mL/g	Viscosity, mPa·s
Gas production parameters			
V, mL/g	0.68^*^	0.67^*^	0.55^*^
V_F_, mL/g	0.64^*^	0.65^*^	0.58^*^
(FRD_0_) × 100, h^−1^	0.09	–0.08	0.11
K, h^−1^	0.37	0.26	0.09
T_1/2_, h	–0.21	–0.01	–0.02
Concentrations of VFAs, mmol/L			
Acetate	0.57^*^	0.61^*^	0.52
Propionate	0.74^**^	0.77^**^	0.44
Butyrate	0.58^*^	0.51	–0.01
Valerate	0.72^**^	0.66^*^	0.18
Total SCFA	0.68^*^	0.68^*^	0.54^*^
Isobutyrate	0.34	0.15	0.45
Isovalerate	0.28	–0.13	0.25
SCFAs/VFAs, %	0.50^*^	0.66^*^	0.40^*^
BCFAs/VFAs, %	–0.59^*^	–0.76^**^	–0.29

*WBC*, water-binding capacity; *WSC*, water swelling capacity; *V*, 48 h cumulative gas production; *V*_*F*_, the final asymptotic gas volume; *FRD*_*0*_, initial fractional rate of degradation at *t*-value = 0; *K*, fractional rate of gas production at a particular time point; *T*_*1/2*_, half-life to asymptote; *VFAs*, volatile fatty acids; total SCFA, the sum of acetate, propionate, butyrate, and valerate; SCFAs/VFAs, the sum of SCFAs (acetate, propionate, butyrate, and valerate)/the sum of VFAs; BCFAs/VFAs, the sum of BCFAs (isobutyrate and isovalerate)/the sum of VFAs; the results in the table are shown as the spearman correlation coefficient. ^*^, ^**^ stand for *P* value < 0.05 and < 0.01, respectively

### Spearman correlations between diarrhea index, ATTD, and SCFAs in colon contents of weaned piglets

Spearman correlation coefficients between diarrhea index, ATTD, and SCFAs in the colon contents of weaned piglets are presented in Table 11. Acetate production was correlated with ATTD of DM, GE, NDF, and ADF (*r* = 0.48, 0.52, 0.39, and 0.43, respectively; *P* < 0.05). Propionate production was correlated with ATTD of DM (*r* = 0.36; *P* < 0.05). Butyrate production was correlated with diarrhea index during d 1–7 and d 1–28, and ATTD of DM, GE, NDF, and ADF (*r* = –0.45, 0.35, 0.51, 0.42, 0.39, and 0.43, respectively; *P* < 0.05). Valerate production was positively correlated with ATTD of DM, NDF, and ADF (*r* = 0.37, 0.36, and 0.56, respectively; *P* < 0.05). Total SCFAs production was positively correlated with ATTD of DM, GE, NDF, and ADF (*r* = 0.61, 0.63, 0.44, and 0.52, respectively; *P* < 0.05).

**Table 11:** Spearman correlations between diarrhea index, apparent total tract digestibility (ATTD), short-chain fatty acid (SCFA), and branched-chain fatty acid (BCFA) concentrations in colon contents of weaned piglets

Spearman correlation			Concentration of SCFAs, µg/g colon content					
	Acetate	Propionate	Butyrate	Valerate	Total SCFA	Isobutyrate	Isovalerate	Total BCFA
Diarrhea index								
D 1 to 7	0.17	0.00	–0.45^**^	–0.15	0.06	–0.07	–0.13	–0.11
D 1 to 14	0.16	–0.07	–0.16	–0.13	0.04	–0.05	–0.11	–0.10
D 1 to 21	0.19	0.04	–0.16	–0.04	0.10	0.04	–0.06	–0.07
D 1 to 28	0.20	–0.17	–0.35^*^	–0.24	0.01	–0.08	–0.12	–0.01
ATTD, %								
DM	0.48^**^	0.36^*^	0.51^**^	0.37^*^	0.61^**^	0.31	0.11	0.20
GE	0.52^**^	0.35	0.42^***^	0.35	0.63^**^	0.31	0.10	0.19
CP	0.07	0.16	0.11	0.13	0.14	0.04	0.12	0.10
NDF	0.39^*^	0.16	0.39^*^	0.36^*^	0.44^*^	0.13	0.05	0.10
ADF	0.43^*^	0.12	0.43^*^	0.56^**^	0.52^**^	0.17	0.21	0.22

*DM* dry matter, *GE* gross energy, *CP* crude protein, *NDF* neutral detergent fiber, *ADF* acid detergent fiber, total SCFA, the sum of acetate, propionate, butyrate, and valerate; total BCFA, isobutyrate and isovalerate; the results in the table are shown as the Spearman correlation coefficient. ^*^, ^**^ stand for *P* value < 0.05 and < 0.01, respectively

## Discussion

Inclusion of specific dietary fibers in weaning diet was reported to alter the intestinal health and nutrient digestion in weaned piglets [3]. For example, dietary fibers with excessive WBC and high viscosity, such as colloidal-derived fiber ingredients, could reduce feeding and digestion in weaned piglets by increasing digesta volume and viscosity [7]. This indicated that understanding the effects of hydration properties of dietary fiber is critical for optimal weaned piglets’ production. In the current trial, we focused on the potential effects of hydration properties of different-source dietary fibers and explored the effects of four dietary fibers with medium or low hydration properties and a low-fiber control diet on the intestinal health and growth performance of weaned piglets. Our results demonstrated that different-source dietary fibers varied in their hydration properties and impacts on diarrhea, microbial composition, and SCFA production in weaned piglets. WB and MCC could exacerbate diarrhea and impair nutrient digestibility, probably because their low hydration properties were detrimental to gut microbial homeostasis and fermentation.

In the previous study, the hydration properties of a dietary fiber were shown to be mainly affected by the structure and composition of fiber polysaccharides [37, 38]. For instance, the water solubility of guar gum increases due to the existence of branched chains in its spatial structure [37]. The medium hydration properties of SBP are related to its composition, with a total dietary fiber content of 600 g/kg, of which 200 g/kg is soluble, mostly pectin, with a high capacity to swell and bind water [39]. MOLP contains polysaccharides with β-1,6 glycosidic linkage, making it soluble and thus a higher WBC [40]. Similarly, *Broussonetia* *papyrifera* leaf powder, mulberry leaf powder, albumen mulberry powder, and mulberry leaf powder are rich in flavonoids and contain a certain amount of polysaccharide, which have a certain water solubility [41]. In this study, the other dietary fibers, such as WB and MCC, showed lower hydration properties due to their high levels of arabinoxylan or insoluble cellulose [42]. Moreover, cereal fiber exhibited lower hydration property than colloidal-derived fiber ingredients, which is consistent with previous literature [37]. The purpose of characterizing these hydration properties is to better understand the role and changes of fibers in the digestion process, due to their effects on some gastrointestinal processes, such as gastric emptying, intestinal motility, or hindgut fermentation. However, the polysaccharide components that affect the hydration properties of these fibers need further identification.

In our previous studies, we have quantified the effects of two available non-traditional dietary fibers with high hydration properties on sow reproductive performance using an in vitro and in vivo method [23, 27, 43]. High hydration fibers were shown to reduce stress and improve intestinal health in pregnant sows [44]. However, unlike sows, 3 to 4-week-old weaned piglets are immature in their potential to digest nutrients (lack of enzymes) and ferment fiber (under-developed microflora) [45]. The dosage and source of dietary fiber need to be carefully considered for weaning diet. Dietary fibers with high WBC and viscosity were reported to concurrently increase stomach distension and reduce effective nutrient digestion and absorption, resulting in lower ADFI and ADG in weaned piglets [9, 46]. However, to our best knowledge, no data are available currently regarding the intervention effect of dietary fibers with different hydration properties on weaned piglets. Based on previous recommendations, the fibrous materials of carrageenan, guar gum, sodium alginate, *Broussonetia* *papyrifera* leaf powder, and mulberry leaf powder may be not suitable for weaning diet due to their excessively high WBC, WSC and viscosity. Therefore, we formulated two dietary fibers with low hydration properties (MCC and WB) and medium hydration properties (MOLP and SBP) in the diets of weaned piglets, and evaluated their in vivo effects on growth performance, diarrhea, diet nutrient digestibility, and intestinal health in weaned piglets. Note that the concentration of a dietary fiber is important, because the intestinal filling caused by high fiber diets could reduce voluntary intake and affect the energy intake of a pig [39]. In this trial, 5% concentration of all the four fibers did not affect the feed intake of piglets, which was consistent with previous studies [47]. Zhao et al. reported that the concentration of 5% WB had beneficial effects on the growth performance and intestinal microflora of weaned piglets [35]. In this trial, the amount of 5% SBP was determined based on the literature review of Kim et al., who mentioned that weaning diets containing 20–80 g/kg NSP could help maintain intestinal barrier function and achieve the maximum growth performance of weaned piglets (5% SBP dietary supplementation, consisting of 20 g/kg insoluble NSP and 10 g/kg soluble NSP) [14].

The digestibility of a dietary fiber is more variable and negatively affected by its amount and source. Just et al. found that every 1.0% of additional crude fiber in a diet could decrease the gross energy digestibility by 1.3% and metabolizable energy by 0.9% [48]. Therefore, the effect of fiber diets on the growth performance and nutrient digestibility of weaned piglets should be considered first. In this study, the four dietary fibers with medium or low hydration properties showed no effect on the growth performance of weaned piglets. Note that both MCC and WB with low hydration properties could consistently reduce the apparent fecal digestibility of DM, GE, ADF and NDF, agreeing with a previous report that dietary fiber provided by wheat by-products had a negative effect on protein digestibility [49]. On the one hand, MCC contains a high content of insoluble fiber and NDF. Insoluble fibers were shown to have low hydration properties and negatively affect the accessibility and action of endogenous enzymes required for insoluble dietary fiber digestion in the upper gut and microbial fermentation in the lower gut [5]. Besides, the impact of NDF fraction on the digestibility coefficient of energy is significant, with approximately 0.1% reduction per 1 g NDF/kg DM [5]. On the other hand, the physicochemical properties of digesta could also affect nutrient digestibility [50]. A rapid passage of digesta may reduce digestion process efficacy. WB is known to relieve of constipation in human subjects [51] and reduce the mean retention time of digesta in the small intestine of pigs [52]. A previous systematic review and meta-analysis of healthy populations found an amount-dependent reduction of 0.78 h in transit time for each additional 1 g of WB per day [53]. A shorter transit time in the digestive tract implies insufficient contact between digestive enzymes and nutrients [54], which can help understand why a low hydration property dietary fiber is detrimental to digestibility. However, the conclusion needs further validation.

Post-weaning diarrhea is considered to be the major reason for reduced nutrient digestibility during the weaning period [55], which may be associated with changes in piglet's intestinal functions. On the one hand, dietary fiber and its metabolites are known to stimulate fluid, electrolyte, and water uptake in colon, thereby reducing diarrhea rates [56]. In this study, DM was 257, 268, 278, 240, and 245 g/kg in colon contents of piglets in CON, MCC, WB, MOLP, and SBP, respectively. Compared with medium hydration property groups, low hydration property groups showed a higher DM content in the piglet colon content. This was consistent with previous reports that SBP or rye could increase the viscosity of small intestinal digesta, leading to a lower DM content of colonic digesta, but had a negligible effect on intestinal health of weaned piglets [57, 58]. Therefore, the reduction of diarrhea rate in this study should not be caused by an increase in chyme DM content. On the other hand, piglets are known to be susceptible to a number of bacterial and viral diseases related to diarrhea at weaning [14]. Gut microbiota disruption is considered as one of the important reasons for post-weaning diarrhea and enteric infections. Therefore, we further evaluated the effects of dietary fibers on the intestinal functions of weaned piglets to explore the underlying mechanisms. The proliferation of beneficial bacteria such as *Lactobacillus*, *Bifidobacterium*, etc. is conducive to improving intestinal health. *Escherichia coli* and *Brachyspira hyodysenteriae* are important pathogenic causes for post-weaning diarrhea, with the former having specific effects on the small intestine and the latter being a pathogen that specifically colonizes the large intestine and causes swine dysentery [14, 59–61]. In this study, compared with medium hydration property groups, low hydration property groups showed lower relative abundances of *Lactobacillus* and *Bifidobacterium*, but higher relative abundances of *Escherichia coli* and qPCR detection rate of *Brachyspira hyodysenteriae*. There are many potential reasons for this difference. On the one hand, the degree of polymerization of dietary fiber has been reported to be the material basis for its promotion on *Bifidobacterium* [62]. The degree of dietary fiber polymerization tends to show a positive correlation with hydration properties [63]. SBP containing a higher degree of polymerization of pectin may be more prone to induce the *Bifidobacterium* growth in vivo. Similar results were also observed in previous studies of pectin was shown to promote the proliferation of microbes such as *Lactobacillus* and *Bifidobacterium* and reduce the abundance of *Brachyspira hyodysenteriae* in weaned piglets [12, 13]. On the other hand, dietary fibers with low hydration properties may inhibit the colonization of beneficial bacteria by damaging the mucous layer [64]. *Bifidobacterium* are known to adhere to intestinal mucins and colonize the mucus layer of the gastrointestinal tract [64]. The flushing effect of dietary fiber with low hydration properties on the intestinal wall may be detrimental to the development of the mucosal layer in weaned piglets. However, the effects of hydration properties on bacteria-fiber interactions need further research to obtain more specific information.

Dietary fiber hydration properties were reported to promote bacterial enzymes from entering the fiber matrix, thus enhancing the degradation and utilization of dietary fibers by microorganisms [11]. The fermentability of dietary fibers may also vary with their hydration properties, so we analyzed the correlation between the hydration properties and fermentation properties of dietary fibers. In this study, dietary fiber in vitro hydration properties were significantly and positively correlated with the production of acetate, propionate, butyrate, and isobutyrate. Besides, the in vivo results showed that the two low hydration property dietary fibers were lower than the two medium hydration property dietary fibers in SCFAs/VFAs, but higher in BCFAs/VFAs. SCFAs have been reported to promote colon growth and probiotic colonization, thus improving gut immune function and gut environment [65]. BCFAs are reliable markers for protein fermentation. Higher protein fermentation has been reported to be associated with increased intestinal epithelial toxicity and post-weaning diarrhea [66]. Further Spearman’s correlation analysis revealed that butyrate may be important metabolites of a dietary fiber affecting weaned piglets, with a negative correlation with diarrhea index and a positive correlation with nutrient digestibility. Therefore, we speculate that one of the mechanisms for medium hydration dietary fibers to regulate intestinal function in weaned piglets may be through their fermentation to produce butyrate. Zhao et al. reported that dietary fibers may enhance the growth performance of weaned piglets by altering gut microbiota and improving butyrate production [35]. Butyrate is particularly important as the most preferred substrate for colon cells, helping maintain their normal phenotype and possessing potential selective antimicrobial effects [67]. Additionally, increased butyrate was considered beneficial to the host by promoting the proliferation of mucosal epithelial cells, the differentiation of intestinal epithelial cells, and the function of colonic barrier [54]. As shown in our in vivo and in vitro results, among the four tested dietary fibers, SBP had the highest capacity to produce butyrate in this study, which could at least partially explain its diarrhea reduction effect. Note that SBP and MOLP with medium hydration properties were not entirely consistent in their effects on weaned piglets in this study, with the latter appearing to be not so beneficial than the former, as reflected by a higher diarrhea rate, abundance of pathogens in colonic contents, and a lower SCFAs level. A possible explanation is that MOLP fermentation could produce more NH_3_-N and increase the colonic pH (NH_3_-N concentration in fermentation broth: 15.8 mmol/L, data not shown), which may be harmful to the intestinal health of weaned piglets. The HCL secretion function of gastric oxyntic cells is not yet perfect in weaned piglets at the early stage of weaning [68]. A higher pH value is considered harmful because it has been shown to increase the numbers of pathogenic bacteria and decrease SCFA production [69]. Moreover, MOLP may produce toxic ammonia–nitrogen metabolites, such as spermidine, histamine, and putrescine, leading to an increased diarrhea rate after weaning [70], thus masking the beneficial effects of fiber fermentability on weaned piglets. This inference needs further validation. Although unable to well explain the phenomenon, the existing results at least indicate that SBP is the most suitable fiber material source for weaned piglets among the four tested fibers.

Post-weaning diarrhea generally induces marked changes in intestinal structure, such as villus atrophy and crypt hyperplasia, resulting in growth lag in weaned piglets [1]. However, in this trial, MCC group with low hydration property and low fermentability showed impairment of small intestinal morphology (lower VCR in jejunum), indicating the poor function and maturity of the intestine. In MCC group, the lower VCR was associated with the increased plasma diamine oxidase. The diamine oxidase, *D*-lactate and endotoxins in serum are useful markers of intestinal permeability. The intestinal epithelial barrier includes a lining of enterocytes and intercellular multiprotein complexes (such as ZO-1 and OCLN), which plays a crucial role in preventing pathogenic bacteria, antigens, and toxins from entering the systemic circulation through the gut lumen. In the present study, MCC supplementation was shown to increase the plasma diamine oxidase activity and reduced the jejunal mRNA levels of *ZO-1* and *OCLN*, suggesting decreased intestinal permeability and intestinal barrier function. Furthermore, gut barrier dysfunction can trigger inflammation response and release a series of inflammation cytokines, a phenomenon also observed in MCC group. Although unable to well explain its negative effects, the existing results at least indicate that MCC is not suitable for weaned piglets as a fibrous materials source.

However, there were some limitations to our study. Firstly, the main physicochemical properties of dietary fiber with nutritional significance are hydration properties, cation exchange capacity, and organic compound absorptive properties [37]. but in the current paper, we only considered hydration properties. Secondly, based on previous studies, dietary fibers with high viscosity or WBC can affect the digestion and absorption of nutrients in weaned piglets [5, 8–10]. We selected only dietary fibers with low and medium hydration properties for in vivo testing, and did not include fibers dietary with higher hydration characteristics, such as guar gum and sodium alginate, in the in vivo tests. Thirdly, in this study, different-source dietary fibers were used as models to mimic different hydration property fibers, and our in vivo test protocol did not guarantee that the other physicochemical properties of the fibers were the same, such as NDF content and polysaccharide composition. We cannot exclude other more subtle differences, indicating the test fibers may not represent all fibers with low or medium hydration properties. Further studies are needed to clearly determine the effect of dietary fiber hydration properties on the growth performance and the intestinal health of weaned piglets. We plan to use different processing methods (sun-drying or tumble-drying), granulation methods (large or small), or the addition of phenolic compounds (dietary fibers have been shown to aggregate and lose viscosity and WBC in solution in the presence of phenolic compounds) to obtain SBP with different hydration properties [6, 71], thus avoiding differences in other properties due to fiber origin.

## Conclusions

Our results demonstrated that different-source dietary fibers vary in their hydration properties and impacts on diarrhea, microbial composition and SCFAs production in weaned piglets. WB and MCC could exacerbate diarrhea and impair nutrient digestibility, probably because their low hydration properties are detrimental to gut microbial homeostasis and fermentation. Notably, SBP showed a positive effect on diarrhea reduction in weaned piglets, probably due to its suitable hydration properties supporting fermentative SCFAs production. Future studies can focus on the effects and mechanisms of other fibrous ingredients with excellent SCFAs production parameters and medium hydration properties on weaned piglets to facilitate the selection of suitable fibrous ingredients in a weaning diet.

## Supplementary Information

**Additional file 1: Table S1. **The inter and intraassay coefficient of variation, and the detection range of the kits. **Table S2.** Primer sequencesfor RT-PCR amplification. 

## Data Availability

The datasets produced and/or analyzed during the current study are available from the corresponding author on reasonable request.

## Abbreviations

ADF: Acid detergent fiber
ADG: Average daily gain
ADFI: Average daily feed intake
ATTD: Apparent total tract digestibility
CF: Crude fiber
CLDN-4: Claudin-4
CON: Control
CP: Crude protein
CSF3: Colony stimulating factor 3
DM: Dry matter
GE: Gross energy
G/F: Gain/feed ratio
IL-1β: Interleukin 1β
IL-10: Interleukin 10
NDF: Neutral detergent fiber
MCC: Microcrystalline cellulose
MOLP: *Moringa oleifera* leaf powder
NSP: Non-starch polysaccharides
OCLN: Occludin
SBP: Sugar beet pulp
SCFA: Short-chain fatty acid
TNF-α: Tumor necrosis factor-α
VCR: Villus height/crypt depth ratio
WB: Wheat bran
WBC: Water binding capacity
WSC: Water swelling capacity
ZO-1: Zonula occludens-1
ZO-2: Zonula occludens-2
