# Supplementary material for: Dietary fibers with low hydration properties exacerbate diarrhea and impair intestinal health and nutrient digestibility in weaned piglets
Source: J Anim Sci Biotechnol. 2022 Nov 9;13:142. doi: 10.1186/s40104-022-00771-7 (PMC9644590; doi:10.1186/s40104-022-00771-7)
Supplement: Supplementary file 1 — Additional file 1: Table S1. The inter and intraassay coefficient of variation, and the detection range of the kits. Table S2. Primer sequencesfor RT-PCR amplification. [file 40104_2022_771_MOESM1_ESM.docx]

**Table S1 The inter and intra assay coefficient of variation, and the detection range of the kits**

| Kits | Coefficient of variation | | Detection range |
| --- | --- | --- | --- |
|  | Inter-assay | Intra-assay |  |
| Diamine oxidase | 8.25% | 10.52% | 1 -100 U/L |
| D-lactate | 12.52% | 10.04% | 1.25 -100 ng/mL |

**Table S1 Primer sequences for RT-PCR amplification**

| Bacteria | Primer sequence, 5′–3′ | Size, bp |
| --- | --- | --- |
| *Universal bacterial reference* | F: GTGSTGCAYGGYYGTCGTCA  R: ACGTCRTCCMCNCCTTCCTC | 147 |
| *Lactobacillus* | F: AGCAGTAGGGAATCTTCCA  R: CGCCACTGGTGTTCYTCCATATA | 176 |
| *Bifidobacterium* | F: GGGTGGTAATGCCGGATG  R: CGAAGGGCTTGCTCCCAGT | 298 |
| *Enterococcus faecium* | F: AGAAATTCCAAACGAACTTG  R: CAGTGCTCTACCTCCATCATT | 92 |
| *Escherichia coli* | F: CATGCCGCGTGTATGAAGAA  R: CGGGTAACGTCAATGAGCAAA | 93 |
| *Salmonella* | F: GCGGCGTTGGAGAGTGATA  R: AGCAATGGAAAAAGCAGGATG | 103 |
| *Brachyspira hyodysenteriae* | F: AGTGAAATAGTTGCTCATATCAAAT  R: GCATCACTGATTAAAGAACCA AT | 122 |
| Gene, pig | Primer sequence, 5′–3′ | Size, bp |
| *β-actin* | F: CACCTTCTACAACGAGCTGC  R: TCATCTTCTCACGGTTGGCT | 95 |
| *Zonula occludens-1* (*ZO-1*) | F: CCAGGGAGAGAAGTGCCAGTAGG  R: TTTGGTGGGTTTGGTGGGTTGAC | 92 |
| *Zonula occludens-2* (*ZO-2*) | F: CCCTCAGCCGTTGCCAGTAATG  R: CTCTCCCACCTCGTCACTCTCTG | 144 |
| *Claudin-4* (*CLDN-4)* | F: TCATCGGCAGCAACATCGTCAC  R: CAGCAGCGAGTCGTACACCTTG | 110 |
| *Occludin* (*OCLN)* | F: CAGTGGTAACTTGGAGGCGT  R: CCGTCGTGTAGTCTGTCTCG | 104 |
| *Tumor necrosis factor-α* (*TNF-α*) | F: GCACTGAGAGCATGATCCGAGAC  R: CGACCAGGAGGAAGGAGAAGAGG | 120 |
| *Interleukin 1β (IL-1β)* | F: AAGAGGGACATGGAGAAGCGATTTG  R: TTGTTCTGCTTGAGAGGTGCTGATG | 114 |
| *Colony stimulating factor 3* (*CSF3*) | F: GACTGGCTGCCTGAACCAACTG  R: TGGTGGCTAAGTCGGTGACATCC | 134 |
| *Interleukin 10 (IL-10)* | F: CTTCGGCCCAGTGAAGAGTT  R: TGCCTTCGGCATTACGTCTT | 154 |
